# Wool-Based Carbon Fiber/MoS_2_ Composite Prepared by Low-Temperature Catalytic Hydrothermal Method and Its Application in the Field of Gas Sensors

**DOI:** 10.3390/nano12071105

**Published:** 2022-03-28

**Authors:** Yidan Xia, Zhaofeng Wu, Zhangjie Qin, Fengjuan Chen, Changwu Lv, Min Zhang, Talgar Shaymurat, Haiming Duan

**Affiliations:** 1 Xinjiang Key Laboratory of Solid State Physics and Devices, Xinjiang University, Urumqi 830046, China; xiayidaaa@163.com (Y.X.); dhm@xju.edu.cn (H.D.); 2 School of Physics Science and Technology, Xinjiang University, Urumqi 830046, China; qinzj0725@163.com (Z.Q.); fjchen@xju.edu.cn (F.C.); lvchw@xju.edu.cn (C.L.); minzhang0816@163.com (M.Z.); 3 Key Laboratory of New Energy and Materials Research, Xinjiang Institute of Engineering, Urumqi 830023, China; talgar.shaymurat@vip.163.com

**Keywords:** WCF–MoS_2_ composite, catalytic hydrothermal method, heterojunction, gas-sensitive properties

## Abstract

Under the background of the Paris Agreement on reducing greenhouse gases, waste wools were converted into wool carbon fiber (WCF) and WCF–MoS_2_ composites by low-temperature catalytic hydrothermal carbonization. Their structures and gas-sensing performances were studied for the first time. Due to the existence of heterojunctions, the responses of the WCF–MoS_2_ composite to the five analytes were 3–400 times those of MoS_2_ and 2–11 times those of WCF. Interestingly, because of the N, P, and S elements contained in wools, the WCF prepared by the hydrothermal method was realized the doping of N, P, and S, which caused the sensing curves of WCF to have different shapes for different analytes. This characteristic was also well demonstrated by the WCF–MoS_2_ composite, which inspired us to realize the discriminative detection only by a single WCF–MoS_2_ sensor and image recognition technology. What’s more, the WCF–MoS_2_ composite also showed a high sensitivity, a high selectivity, and a rapid response to NH_3_. The response time and the recovery time to 3 ppm NH_3_ were about 16 and 5 s, respectively. The detection of limit of WCF–MoS_2_ for NH_3_ was 19.1 ppb. This work provides a new idea for the development of sensors and the resource utilization of wool waste.

## 1. Introduction

Since the Paris Agreement aims to limit the rise in global temperature to less than 2 °C, governments have set their own policies to reduce greenhouse gases [1,2]. However, many difficulties still need to be overcome to achieve the goals of the Paris Agreement. We need to consider not only the fossil fuels consumed by economic development, but also more than 100 billion tons of biological waste that we process every year [3,4,5]. The main component of biomass waste, such as plant straw and waste wool, is generally carbon. The general treatment methods for biomass waste, such as incineration and landfilling, produce carbon dioxide or methane gas, accelerating the rise in global temperature [6,7]. Even if biomass waste is processed into feeds and fertilizers, these feeds and fertilizers will eventually return to the atmosphere in the form of carbon dioxide after being consumed by animals and plants [2,8,9]. Therefore, there is an urgent need for new methods and technologies to deal with biomass waste and prevent or reduce the carbon in biomass from returning to the atmosphere in the form of carbon dioxide or methane.

Take wool as an example. There are more than four million tons of waste wool clothes, inferior wool, and wool byproducts from textile mills that are not available every year [10,11]. The main component of wool is keratin, which is mainly composed of C, H, O, N, S, and P elements, of which the content of C accounts for about 50% [12,13]. From the perspective of reducing greenhouse gas emissions, converting organic biomass carbon into inorganic biomass carbon materials (BCMs) through hydrothermal carbonization or direct carbonization is a feasible strategy [4,5,14]. On the one hand, as semiconductor materials, BCMs have attracted more and more attention because of their rich raw materials, diverse morphology, adjustable properties, good stability, and low price [6,14,15]. On the other hand, either direct carbonization or hydrothermal carbonization can convert biomass waste into solid inorganic BCMs that can be stored for a long time [4,5,16]. This transformation not only is convenient and economically feasible, but can also effectively prevent the carbon in biomass from returning to the atmospheric environment in the form of greenhouse gas [4,16,17]. Therefore, BCMs have developed rapidly in recent years and have been applied more and more to the fields of energy storage, electromagnetic shielding, carbon dioxide adsorption, oil–water separation, gas sensing, etc. [6,18,19,20]. Generally, the temperature of direct carbonization is 500–1000 °C, while the common temperature of hydrothermal carbonization is 180–250 °C [14,17]. Generally speaking, biomass wastes are rich in functional groups. However, due to the high temperature of direct carbonization, there are very few functional groups on the surface of BCMs prepared by direct carbonization [6]. In sharp contrast, due to the lower temperature of hydrothermal carbonization, BCMs prepared by hydrothermal carbonization have rich functional groups, which play an important role in the fields of energy storage, gas adsorption, and gas sensing [6,21]. In addition, in order to prevent the oxidation of biomass and improve the carbon yield, the preparation of BCMs by direct carbonization often needs to be carried out in a vacuum environment or under the protection of inert gas [6,22]. From here, we see that hydrothermal carbonization has the advantages of rich functional groups, low temperature, low power consumption, and simple equipment. Furthermore, hydrothermal carbonization is also convenient for adding a catalyst in an aqueous medium, which can not only promote the carbonization of biomass at a low temperature but achieve the doping of BCMs [14,23]. These characteristics of hydrothermal carbonization are very favorable for transforming biomass waste into solid inorganic BCMs with low power consumption and regulating properties.

Herein, in order to prevent the carbon in waste wool from returning to the atmospheric environment in the form of greenhouse gas as well as realizing its resourceful utilization, we creatively used waste wool as a carbon source and a MoS_2_ precursor as a catalyst to prepare wool-based carbon fiber (WCF)–MoS_2_ composites by low-temperature hydrothermal carbonization. Meanwhile, trace elements in wool, such as N, S, and P, were used to realize the atomic doping of WCF, so as to adjust its structure and properties. Then, a WCF–MoS_2_ composite with heterojunctions was prepared as a gas sensor for the first time, and its potential as a gas-sensing material was studied by comparing the gas-sensing properties of the WCF–MoS_2_ composite with those of MoS_2_ and WCF. In addition, we also analyzed the effect of catalytic hydrothermal carbonization on the microstructure of wool, in order to provide a useful reference for the hydrothermal carbonization of other wool analogues. This is the first comparative study on the gas-sensitive properties of MoS_2_, WCF, and WCF–MoS_2_ composites.

## 2. Materials and Methods

### 2.1. Materials

Ammonium molybdate ((NH_4_)_6_Mo_7_O_24_·4H_2_O) and thiourea (CH_4_N_2_S) were analytical reagents, purchased from Sinopharm Chemical Reagent Co., Ltd. The wool came from sheep in Bole City, Xinjiang Uygur Autonomous Region, China.

#### 2.1.1. Preparation of MoS_2_

Typically, 1 mmol (NH_4_)_6_Mo_7_O_24_·4H_2_O and 30 mmol CH_4_N_2_S were dissolved in 35 mL of deionized water and stirred vigorously to form a homogeneous solution. This solution was put into a 50 mL Teflon-lined stainless steel autoclave and maintained at 120 °C for 10 h. After the reactor was cooled to room temperature, the product was dried and labeled as MoS_2_.

#### 2.1.2. Preparation of WCF and WCF–MoS_2_ Composites

Of the wool collected, 0.50 g was repeatedly rinsed with deionized water and ethanol, and it was placed in a 50 mL Teflon-lined stainless steel autoclave with 35 mL of deionized water and maintained at 120 °C for 10 h. After the reactor was cooled to room temperature, the product was dried and labeled as WCF. Of the wool, 0.50 g was put together with 1.15 g of (NH_4_)_6_Mo_7_O_24_·4H_2_O, 1.52 g of CH_4_N_2_S and 35 mL of deionized water in a 50 mL stainless steel autoclave lined with PTFE, sealed and kept at 120 °C for 10 h. After the reactor was cooled to room temperature, the product was dried and labeled as WCF–MoS_2_.

### 2.2. Device Fabrication and Testing

A certain amount of sensing material was mixed with deionized water, then ground into a paste and coated onto an interdigital electrode. Then, the interdigital electrode was dried at 25 °C and aged for 24 h with a voltage of 4 V to obtain a sensing chip with good stability. The target vapor was produced by thermal evaporation, according to Equation (1):

Q = (V × C × MW)/(22.4 × d × ρ) × 10^−9^ × (273 + T_R_)/(273 + T_C_), (1)

where Q and V are the volume of the liquid to be taken and the volume of the test container, respectively; MW is the molecular weight of the substance; d is the purity of the liquid; C is the concentration of the gas to be prepared; ρ is the density of the liquid; and T_R_ and T_C_ are the ambient temperature and the temperature in the test container, respectively [6]. An electrochemical workstation (CIMPS-2, ZAHER EMNIOM) was used to record sensing signals at room temperature. During the gas-sensing test, a voltage of 4 V was applied to both ends of the sensing chip. The response was defined as: $\mathrm{Response}=\left(\frac{I_{G}-I_{R}}{I_{R}}\right)\times 100\%$, where I_R_ and I_G_ are the currents of the sensor in the reference gas and target gas, respectively [24]. The response time and recovery time are defined as the response values of 90% and 10% of the current of the sensor in contact with the target gas, respectively.

### 2.3. Materials Characterization

The composition, structure, and morphology of samples were studied by means of field-emission scanning electron microscopy (FE-SEM; S-4800; Hitachi, Japan), mapping (Mapping; S-4800, Hitachi, Japan), XRD (Bruker D8 Advance, with Cu-Kα radiation), Raman spectroscopy (SENTERRA Compact Raman Microscope, Instrument: LabRAM HR Evol, Acq; time: 180 s, laser: 785 nm_Edge, objective: ×10_VIS, range (cm^−1^): 100–3200), and XPS (USA Thermo Fisher-Thermo SCIENTIFIC ESCALAB 250Xi, MA, Massachusetts, USA). The band structure and surface functional groups of samples were measured by ultraviolet photoelectron spectroscopy (UPS; USA Thermo Fisher-Thermo Fisher Nexsa, MA, USA) and FT-IR (Bruker VERTEX70, Germany 4.0 cm^−1^ SCANS 200, KA, Karlsruhe, Germany).

## 3. Results and Discussion

### 3.1. Structures and Morphologies of MoS_2_, WCF, and WCF–MoS_2_

**Figure 1:**
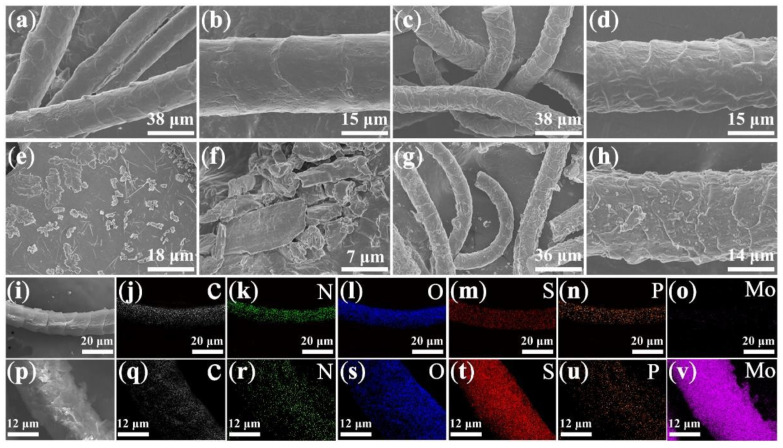
SEM images at different magnifications of wool fiber (**a**,**b**), wool-based carbon fiber (WCF) (**c**,**d**), MoS_2_ (**e**,**f**), WCF–MoS_2_ (**g**,**h**); element mapping of WCF (**i**–**o**) and WCF–MoS_2_ composites (**p**–**v**).

Figure 1 shows the SEM images of the wool fiber, WCF, MoS_2_, and WCF–MoS_2_. As shown in Figure 1a–d, both the wool fiber and WCF were fibrous, with a diameter of about 15 μm. Meanwhile, wrinkles appeared on the surface of the wool fiber and the WCF, but the wrinkles on the WCF surface were denser, indicating that hydrothermal carbonization changed the microstructure of the wool fiber. Pure MoS_2_ was granular (Figure 1e,f), and the WCF–MoS_2_ composite was still fibrous, with a diameter of about 15 μm (Figure 1g,h). Moreover, there were still wrinkles on the surface of the WCF–MoS_2_ fiber, but the surface became coarser due to the nanoparticles loaded on the fiber [25]. This showed that WCF was coated by a thin layer of MoS_2_ to form the WCF–MoS_2_ composite, which was also proven by the element mapping. As shown in Figure 1i–o, WCF uniformly contained C, N, S, O, and a small amount of P elements. Compared with WCF, the WCF–MoS_2_ composite not only contained more S, but also contained Mo, which indicated that WCF was indeed coated with a layer of MoS_2_ to form a uniform WCF–MoS_2_ composite.

**Figure 2:**
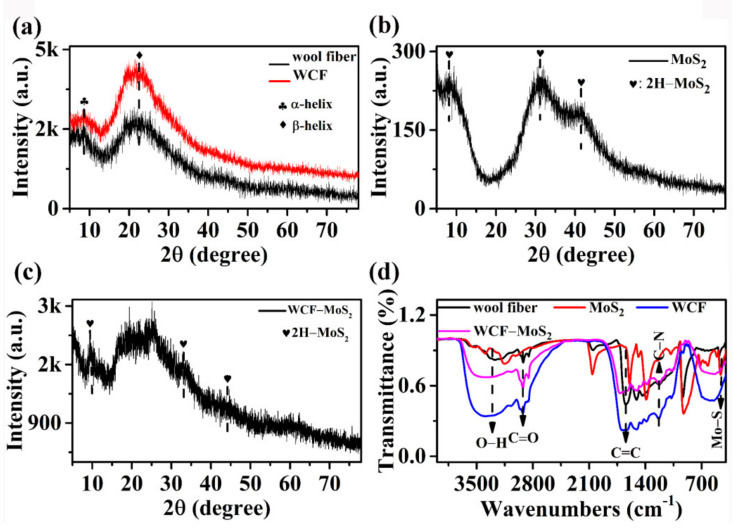
XRD patterns of wool fiber and WCF (**a**), MoS_2_ (**b**), and WCF–MoS_2_ (**c**); (**d**) FT–IR spectra of wool fiber, WCF, MoS_2_, and WCF–MoS_2_.

The structures of wool fiber, WCF, MoS_2_, and the WCF–MoS_2_ composite were studied using an XRD analysis. It can be clearly seen from Figure 2a that there were two peaks at 9.0° and 23.6° for wool fiber and WCF, which corresponded to the α-helix and β-sheet structures of the protein, respectively [26,27]. This is consistent with the fact that wool is composed of protein. Compared with that of the wool fiber, the structure of WCF did not change significantly after the hydrothermal treatment. In Figure 2b, we can see that 2H-MoS_2_ peaks (JCPDS Card No. 73-1508) appeared at 9.9°, 32.3°, and 43.2°, which are consistent with previous reports [24,28]. In Figure 2c, the characteristic peaks of 2H MoS_2_ and the wool fiber also appeared in the WCF–MoS_2_ composite. This indicates that the precursor of MoS_2_ may promote the carbonization and structural transformation of wool fiber during the hydrothermal process.

In order to evaluate the effect of the hydrothermal process on the samples, the functional groups of the samples were characterized by the FTIR spectra (Figure 2d). For wool fiber and WCF, there was a C=C extension absorption peak at 1641 cm^−1^ and an O–H stretching vibration at about 3300 cm^−1^ [29]. For MoS_2_, Mo–S bonds at 459 cm^−1^ showed that MoS_2_ was successfully prepared [30]. For WCF–MoS_2_, the peaks at 3297 and 2853 cm^−1^ corresponded to O–H and C=O groups, respectively [31]. The peak at 1640 cm^−1^ belonged to the C=C stretching vibration. At the same time, Mo–S groups appeared near 525 cm^−1^, indicating that MoS_2_ was loaded on the surface of WCF–MoS_2_ [32].

**Figure 3:**
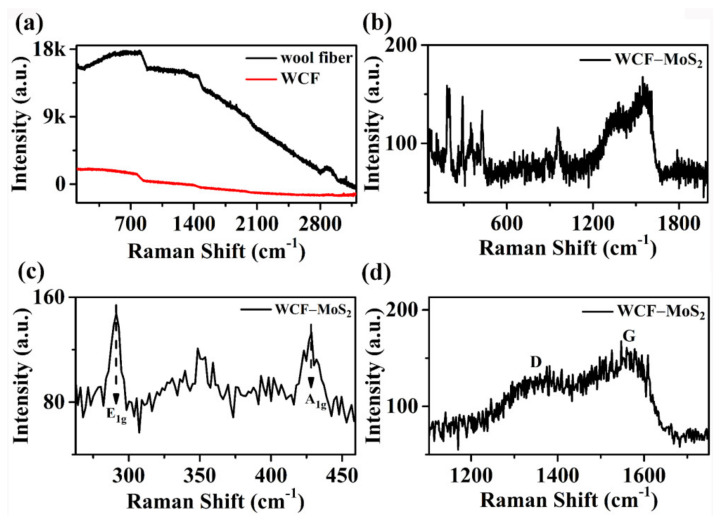
Raman spectra of wool fiber and WCF (**a**) and WCF–MoS_2_ (**b**–**d**).

The carbon structure and graphitization degree were studied by Raman spectroscopy (Figure 3). As shown in Figure 3a, the Raman spectra of wool fiber and WCF did not change significantly, but neither of them showed obvious G and D bands characteristic of carbon materials [23]. This indicated that the simple low-temperature hydrothermal treatment may only carbonize the surface of the wool fiber, so it did not show obvious peaks characteristic of carbon [23]. For the WCF–MoS_2_ composite, the Raman spectra showed the bands characteristic of MoS_2_ and carbon materials (Figure 3b). Figure 3c is the Raman spectrum of WCF–MoS_2_ in the range of 270 to 455 cm^−1^. Figure 3c shows that the low-energy region consisted of various first-order modes, namely A_1g_ (430 cm^−1^) and E_1g_ (287 cm^−1^) of MoS_2_ [26]. Defective bands usually occur in the spectral range of 140–420 cm^−1^ in MoS_2_ [33]. Figure 3d is the Raman spectrum of the WCF–MoS_2_ composite in the range of 1100 to 1750 cm^−1^. It is worth noting that the D (1352 cm^−1^) and G (1579 cm^−1^) bands corresponding to carbon appeared for the WCF–MoS_2_ composite. This indicated that the precursor of MoS_2_ may play a catalytic role in the hydrothermal process and promote the carbonization of wool, forming a WCF–MoS_2_ composite [6].

**Figure 4:**
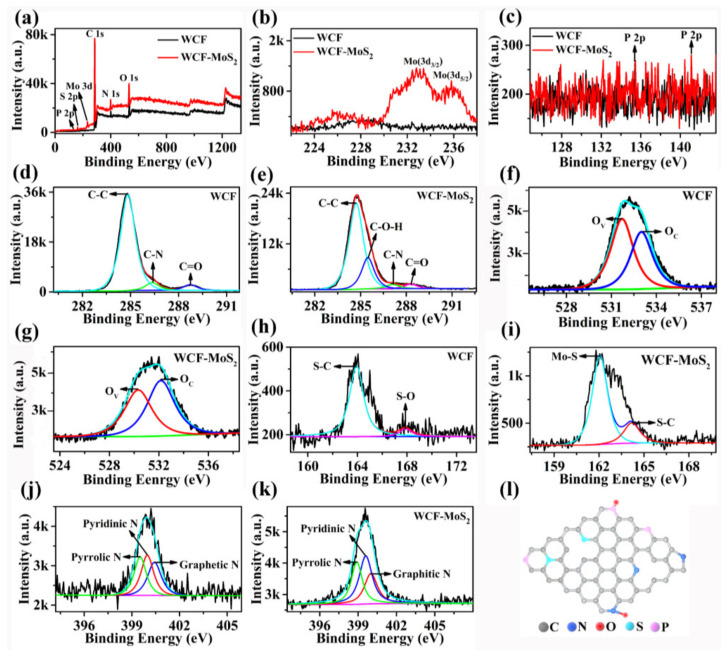
(**a**) XPS spectra of WCF and WCF–MoS_2_. High-resolution spectra of Mo 3d (**b**), P 2p (**c**), C 1s of WCF (**d**), C 1s of WCF–MoS_2_ (**e**), O 1s of WCF (**f**), O 1s of WCF–MoS_2_ (**g**), S 2p of WCF (**h**), S 2p of WCF–MoS_2_ (**i**), N 1s of WCF (**j**), N 1s of WCF–MoS_2_ (**k**). (**l**) Schematic diagram of highly defective graphene formed on the surface of WCF.

XPS was also used to analyze the elements and structure of samples. As can be seen from Figure 4a, both WCF and the WCF–MoS_2_ composite contained C, N, O, S, N, and P elements. For the WCF, the proportions of C, N, O, P, S, and Mo in total elements were 85.11%, 3.30%, 10.33%, 0.31%, 0.83%, and 0.13%, respectively. For the WCF–MoS_2_ composite, the proportions of C, N, O, P, S, and Mo elements in the total elements were 78.4%, 8.36%, 9.03%, 0.55%, 2.63%, and 1.02%, respectively. Compared with those of the WCF, the S and Mo elements of WCF–MoS_2_ were significantly increased, which should be derived from the loaded MoS_2_. It can be clearly seen in Figure 4b that two peaks of Mo 3d_3/2_ and Mo 3d_5/2_ appeared at the positions of 232.4 and 235.5 eV, respectively [34,35,36]. However, the peaks corresponding to Mo were not obvious, which indicated that there was only a thin layer of MoS_2_ on the surface of the WCF. The contents of P element in the WCF and the WCF–MoS_2_ composite were only 0.31% and 0.55%, respectively, so their peaks were not very obvious (Figure 4c). Figure 4d shows the C 1s peak of the WCF, and the peaks at 285.1, 286.5, and 288.9 eV represented C–C, C–N, and C=O functional groups, respectively. Figure 4e shows the C 1s peak of WCF–MoS_2_, and the peaks at 285.1, 286.1, 287.2, and 288.9 eV represented C–C, C–O–H, C–N, and C=O functional groups, respectively. On the whole, among the peaks of all the elements, the peak of carbon was the strongest, which indicated that the main element of WCF and the WCF –MoS_2_ composite is carbon. Figure 4f,g show the peaks of O 1s in WCF and the WCF–MoS_2_ composite, respectively. O_V_ and O_C_ were oxygen vacancies and chemisorbed oxygen species, respectively [37]. The percentages of O_C_ in the total oxygen element area in WCF and WCF–MoS_2_ were 45.6% and 55.7%, respectively. This indicated that the percentage of O_C_ in WCF–MoS_2_ was much higher than that in WCF, which may be due to the adsorption of oxygen promoted by the heterojunction of WCF and MoS_2_. Figure 4h shows the S 2p peak of WCF, and the two peaks at 164.2 and 167.9 eV were S–C and S–O functional groups, respectively. For the S 2p peak of WCF–MoS_2_ in Figure 4i, the two peaks at 163.6 and 167.9 eV should be S in MoS_2_ [38], and the latter may be the S of amino acids. Figure 4j shows the N 1s peak of WCF, and there were three peaks at 398.9, 399.5, and 400.1 eV, corresponding to pyrrolic N, pyridinic N, and graphite N, respectively [39]. Similarly, there were three peaks at 399.1, 399.6, and 400.1 eV, corresponding to pyrrolic N, pyridinic N, and graphite N of WCF–MoS_2_, respectively (Figure 4k). Thus, according to the analysis of XPS and Raman spectroscopy, the basic surface structures of WCF and the WCF–MoS_2_ composite should be graphene with high defects (such as the doping of multiple atoms and vacancy), as shown in Figure 4l [40]. Compared with perfect graphene, this defective graphene provided more active sites for gas adsorption, thus achieving the purpose of improving gas sensitivity.

### 3.2. Sensing Performance of MoS_2_, WCF, and WCF–MoS_2_

**Figure 5:**
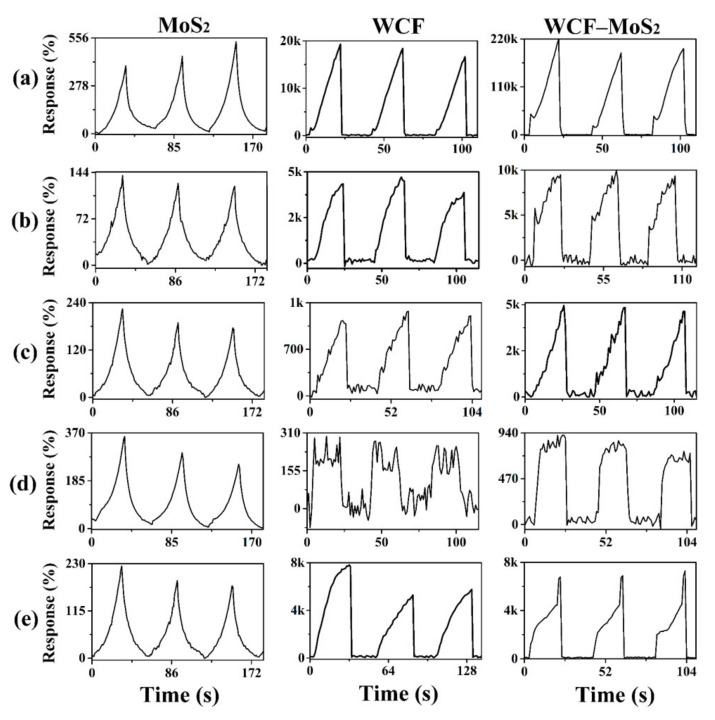
Sensing curves of MoS_2_, WCF, and WCF–MoS_2_ to 1000 ppm of NH_3_ (**a**), the 85% relative humidity (RH) (**b**), 1000 ppm of HCHO (**c**), 1000 ppm of C_3_H_6_O (**d**), and 1000 ppm of C_2_H_5_OH (**e**).

The sensing curves of the sensors based on MoS_2_, WCF, and WCF–MoS_2_ for an 85% relative humidity (RH) and 1000 ppm of NH_3_, formaldehyde (HCHO), acetone (C_3_H_6_O) and ethanol (C_2_H_5_OH) are shown in Figure 5. On the whole, the sensing curves of MoS_2_, WCF, and WCF–MoS_2_ for five target analytes showed they had good recovery abilities and reproducibilities in three consecutive sensing cycles (Figure 5). It is worth noting that the shapes of the sensing curves of MoS_2_ for the five atmospheres are very similar, and the responses were small, which were no more than 459%. Compared with those of MoS_2_, the shapes of the sensing curves of WCF for the five analytes are obviously different, and the responses were greatly improved. Among them, WCF had the highest response to NH_3_, up to 18.67k%. For the five analytes, the different shapes of the sensing curves should be attributed to the fact that WCF had a variety of heteroatom doping (N, P, and S) and rich functional groups [41]. For the WCF–MoS_2_ composite, the sensing curves for five target atmospheres also have different shapes, and the shapes are similar to those of WCF. This showed that the WCF in the WCF–MoS_2_ composite played an important role in the gas-sensing process. For different target atmospheres, the sensing curves of different shapes may be used as fingerprints, which can be combined with databases and image recognition to achieve the effect of discriminative detection. Moreover, both the sensitivity and selectivity of WCF–MoS_2_ were further improved. Its response to NH_3_ reached 213.03k%; the improvement of selectivity and sensitivity should be attributed to the heterojunction structure of the WCF–MoS_2_ composite.

**Figure 6:**
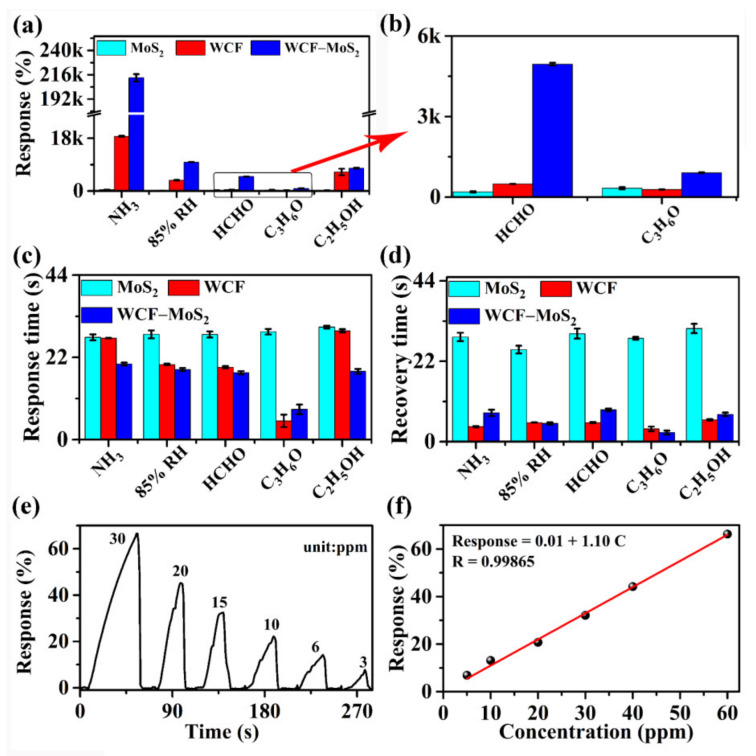
(**a**) Average responses; (**b**) enlarged part of (**a**); (**c**) response times; and (**d**) recovery times corresponding to the sensing curves for MoS_2_, WCF, and the WCF–MoS_2_ composite; (**e**) sensing curves of WCF–MoS_2_ to different concentrations of NH_3_; (**f**) fitting plots of the response vs. the concentration of NH_3_.

Figure 6 shows the average response, response time, and recovery time of MoS_2_, WCF, and WCF–MoS_2_ for the target analyte. As can be seen from Figure 6a,b, the sensitivities of WCF–MoS_2_ to the five analytes were significantly higher than those of MoS_2_ and WCF. For MoS_2_, its responses to NH_3_, the 85% RH, HCHO, C_3_H_6_O, and C_2_H_5_OH were only 459%, 137.3%, 1.432k%, 332%, and 216%, respectively. For WCF, its responses to NH_3_, the 85% RH, HCHO, C_3_H_6_O, and C_2_H_5_OH were up to 18.67k%, 3.673k%, 492.6%, 284%, and 6.482k%, respectively. For the WCF–MoS_2_ composite, its responses to NH_3_, the 85% RH, HCHO, C_3_H_6_O, and C_2_H_5_OH were up to 213.03k%, 9.854k%, 4.954k%, 904.5%, and 7.796k%, respectively. It is can be seen that the response to 1000 ppm of NH_3_ increased from 459% for MoS_2_ to 18.67k% for WCF and to 213.03k% for WCF–MoS_2_. Thus, compared with WCF and MoS_2_, the WCF–MoS_2_ composite showed higher sensitivity and selectivity, which is very important for anti-interference and the highly sensitive detection of NH_3_. As shown in Figure 6c, among the three sensing materials, the response time of the WCF –MoS_2_ composite was basically the shortest, and the response times to the five analytes did not exceed 20 s. The recovery time of the WCF–MoS_2_ composite did not exceed 9 s, which was much lower than that (~25 s) of MoS_2_ (Figure 6d). This means that the WCF–MoS_2_ composite can complete a response–recovery cycle in 30 s and realize the real-time monitoring of NH_3_. NH_3_ is a widely distributed and harmful gas emitted by most chemical industries as well as agricultural production. Prolonged exposure to high concentrations of NH_3_ can damage the eyes, skin, and respiratory organs [42]. The Occupational Safety and Health Administration (OSHA) reported that the human body’s exposure to NH_3_ should not exceed 25 ppm over 8 h. Therefore, the limit of detection (LoD) for NH_3_ based on the WCF –MoS_2_ composite was further evaluated. Figure 6e shows the sensing curves of the WCF–MoS_2_ composite to different concentrations of NH_3_. The WCF–MoS_2_ composite could actually detect 3 ppm of NH_3_, and its response time and recovery time were only 16 and 5 s, respectively. It can be seen from Figure 6f that there was a good linear relationship between the responses and concentrations of NH_3_. The estimated LoD (defined as LoD = 3 S_D_/m, where m is the slope of the linear part of the calibration curve and S_D_ is the standard deviation of noise in the sensing curve) for WCF –MoS_2_ to NH_3_ was determined to be 19.1 ppb.

In order to comprehensively evaluate the gas-sensing performance of WCF-MoS_2_, the performances of recent NH_3_ sensors are compared in Table 1. As shown in Table 1, the response time and recovery time of WCF–MoS_2_ for NH_3_ were 16 and 5 s, respectively, which were close to the shortest response time (14 s) and recovery time (4 s) shown by ZnO/CMTs [37]. Moreover, the LoD (19.1 ppb) of WCF–MoS_2_ could not catch up with the LoD (9 ppb) of ZnO/CMTs. Nevertheless, compared with the preparation temperature (600 °C) of ZnO/CMTs, the preparation temperature of WCF–MoS_2_ was only 120 °C, and the preparation equipment was also simpler. Although the LoD of SnO_2_/Pd/RGO to NH_3_ is only 11 ppb, the response time and recovery time are up to 7 and 50 min, which are not suitable for the real-time monitoring of NH_3_ [43]. Therefore, the WCF–MoS_2_ sensor showed a good comprehensive sensing performance, which not only had a low preparation temperature, a low working temperature, a high sensitivity, and a high selectivity, but also has a fast response and recovery ability.

Furthermore, the discriminative detection of a target atmosphere is still a challenging problem. In order to realize the discriminative detection of a target atmosphere, two methods are generally used, that is, improving the selectivity of sensors and building sensor arrays [6,51]. This requires the screening and subsequent modification of sensing materials, which increases the cost and prolongs the preparation cycle. Because the WCF–MoS_2_ sensor produces sensing curves with different shapes for the five analytes, it inspires us to use image recognition technology to help improve the discriminative detection of a target atmosphere. When the WCF–MoS_2_ sensor is tested for five atmospheres, the shapes of response–recovery curves are saved to form a database. When the WCF–MoS_2_ sensor encounters a certain atmosphere again, new sensing curves will be generated. These new sensing curves will be compared with the database, and the discriminative results will be given. In this way, with the aid of image recognition technology, the discriminative detection of a variety of single atmospheres can be realized by only a single sensor. According to Figure S1, we have preliminarily realized the discriminative detection of five target analytes with the help of MATLAB software and image recognition technology. Due to the small sample capacity, the recognition accuracies of the five target analytes were as high as 100%, proving that our scheme is feasible. This may provide a new scheme and idea for the development of gas sensors.

### 3.3. Possible Sensing Mechanisms of WCF–MoS_2_

**Figure 7:**
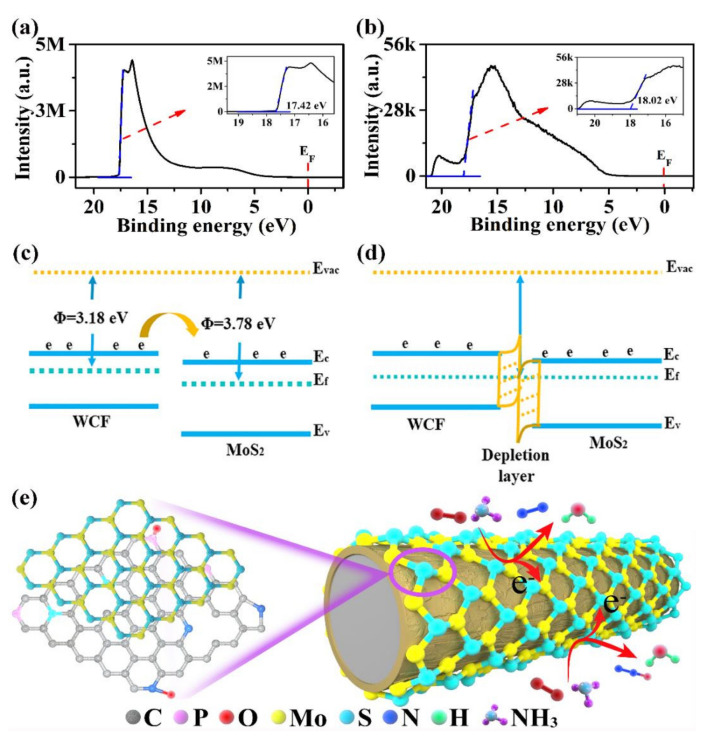
UPS spectra: (**a**) WCF; (**b**) MoS_2_. Energy band structures: (**c**) WCF and MoS_2_; (**d**) the WCF–MoS_2_ composite. (**e**) Sensing mechanism of the WCF–MoS_2_ composite to NH_3_.

In order to analyze the possible sensing mechanism, UPS was performed (Figure 7a,b). Figure 7a,b show the UPS spectra of WCF and MoS_2_, where E_f_ and Φ are the Fermi levels and the working function, respectively. The Φ values of WCF and MoS_2_ were calculated to be 3.18 and 3.78 eV, respectively. Due to the higher E_f_ of WCF than that of MoS_2_, when they come into contact, electrons can be quickly transferred from WCF to MoS_2_. Thus, the bands of WCF and MoS_2_ began to bend, until their Fermi levels reached a new equilibrium (Figure 7c,d), forming a built-in barrier (qV_0_). Therefore, a depletion layer was formed at the interface between WCF and MoS_2_ [52,53]. When the WCF–MoS_2_ composite was exposed to the air, the O_2_ molecules of the air were adsorbed on the surface of WCF–MoS_2_ and captured electrons from the WCF–MoS_2_ composite to form negative oxygen ions, as follows [37]:

(2) $$O_{2}\left(\mathrm{ads}\right)+e^{-}\to O_{2}^{-}\;(<100\;{}^{\circ}C).$$

According to the XPS analysis, the O_2_ molecules adsorbed on the surface of WCF–MoS_2_ were 10% more than those absorbed on the surface of WCF. Thus, O_2_ molecules capture electrons in WCF–MoS_2_ to form a new barrier ($qV_{1}$), which increased the total barrier ($q\left(V_{0}+V_{1}\right)$). Taking the gas sensing of the WCF–MoS_2_ composite to NH_3_ as an example. When the WCF–MoS_2_ composite was in NH_3_ gas, some NH_3_ molecules were absorbed on the surface and reacted with $O_{2}^{-}\;\left(\mathrm{ads}\right)$. The reaction of NH_3_ molecules with $O_{2}^{-}\;\left(\mathrm{ads}\right)$ can be described as follows [37]:

(3) $$2{\mathrm{NH}}_{3}+\left(\frac{3}{2}\right)O_{2}^{-}\;\left(\mathrm{ads}\right)\to H_{2}O+N_{2}+3e^{-},$$

(4) $$2{\mathrm{NH}}_{3}+2O_{2}^{-}\;\left(\mathrm{ads}\right)\to H_{2}O+N_{2}O+2e^{-}.$$

The reaction between NH_3_ molecules and O^2−^ ions (Figure 7e) released electrons into WCF–MoS_2_ and lowered the potential barrier ($q\left(V_{0}+V_{1}-V_{2}\right)$). The resistance of semiconductor materials is reported to be exponentially proportional to the height of the potential barrier [54]:

(5) $$R=R_{0}e^{\frac{\mathrm{qV}}{\mathrm{KT}}},$$

where q*V* is the effective potential barrier, R_0_ is a constant, K is Boltzmann’s constant, and T is the absolute temperature. It is observed that a slight change in the potential barrier will result in a significant change in the sensing resistance, producing an obvious sensing signal. It can be seen that the MoS_2_ precursor plays a catalytic role in the carbonization of wool fiber and forms a heterojunction structure, which promotes the gas-sensing performance of the WCF–MoS_2_ composite.

## 4. Conclusions

WCF and a WCF–MoS_2_ composite were successfully prepared by low-temperature (120 °C) hydrothermal carbonization. It was found that a MoS_2_ precursor played a catalytic role in the carbonization of wool fiber, forming heterojunctions between MoS_2_ and WCF in the WCF–MoS_2_ composite. Compared with MoS_2_ and WCF, the WCF–MoS_2_ composite showed a better gas-sensing performance to NH_3_ due to heterojunctions. Meanwhile, the WCF–MoS_2_ composite showed a good comprehensive sensing performance, which not only had a low preparation temperature, a low working temperature, a high sensitivity (19.1 ppb), and a high selectivity, but also had a fast response–recovery ability (< 30 s). Interestingly, the WCF–MoS_2_ composite also displayed different shapes of sensing curves for different atmospheres, providing a new possibility for the discriminative detection of the target analytes. In response to global warming, this work provides a new idea for the low-temperature preparation of carbon materials, the development of sensors, and the resource utilization of wool waste.

## Figures and Tables

**Table 1:** Sensing performances of the recently reported NH_3_ sensors.

Materials	Concentration (ppm)	Temperature (°C)	Response (%)	Response Time (s)	Recovery Time (s)	Limit of Detection (LoD; ppb)	Reference
Py-rGO	50	200	-	134	310	-	[44]
SnO_2_	1055	350	-	9	37	-	[45]
SnO_2_/Pd/RGO	5	RT	7.6	25,200	3000	-	[43]
ZnO/SiO_2_	50	RT	-	-	-	-	[46]
CuS	-	-	-	55	43	-	[47]
3D RGO/PANI	50	RT	11	370	675	-	[48]
α-Fe_2_O_3_/graphene	10	250	13.5	152	648	-	[42]
rGO-WS_2_	10	33.5	121	60	300	-	[49]
ZIF-67/rGO	50	RT	477	46.4	66.5	74	[50]
CMTs	50	RT	362	16	4	63	[37]
ZnO/CMTs			2091	14	4	9	
WCF-MoS_2_	3	RT	8	16	5	19.1	Present work

## Data Availability

The data presented in this study are available on request from the corresponding author.

## Supplementary Materials

The following supporting information can be downloaded at: https://www.mdpi.com/article/10.3390/nano12071105/s1, Figure S1: Identification of (a) NH_3_, (b) 85% RH, (c) HCHO, (d) C_3_H_6_O and (e) C_2_H_5_OH target analytes by MATLAB software, (f) Curve of training times and accuracy.

## References

[1] Schleussner C.-F., Rogelj J., Schaeffer M., Lissner T., Licker R., Fischer E.M., Knutti R., Levermann A., Frieler K., Hare W. (2016). Science and policy characteristics of the Paris Agreement temperature goal. Nat. Clim. Chang。.

[2] Holden P.B., Edwards N.R., Ridgwell A., Wilkinson R.D., Fraedrich K., Lunkeit F., Pollitt H.E., Mercure J.-F., Salas P., Lam A. (2018). Climate–carbon cycle uncertainties and the Paris Agreement. Nat. Clim. Chang。.

[3] Cho E.J., Trinh L.T.P., Song Y., Lee Y.G., Bae H.-J. (2019). Bioconversion of biomass waste into high value chemicals. Bioresour。 Technol..

[4] Cheng F., Porter M.D., Colosi L.M. (2020). Is hydrothermal treatment coupled with carbon capture and storage an energy-producing negative emissions technology?. Energy Convers. Manag..

[5] Titirici M.-M., Thomas A., Antonietti M. (2007). Back in the black: Hydrothermal carbonization of plant material as an efficient chemical process to treat the CO_2_ problem?. New J. Chem..

[6] Cao S., Wu Z., Sun Q., Zhang W., Beysen S., Wang S., Shaymurat T., Zhang M., Duan H. (2021). Gas sensing properties of cotton-based carbon fibers and ZnO/carbon fibers regulated by changing carbonization temperatures. Sens. Actuators B Chem..

[7] Alatzas S., Moustakas K., Malamis D., Vakalis S. (2019). Biomass Potential from Agricultural Waste for Energetic Utilization in Greece. Energies.

[8] Foong S.Y., Liew R.K., Yang Y., Cheng Y.W., Yek P.N.Y., Mahari W.A.W., Lee X.Y., Han C.S., Vo D.-V.N., Le Q.V. (2020). Valorization of biomass waste to engineered activated biochar by microwave pyrolysis: Progress, challenges, and future directions. Chem. Eng. J..

[9] Yi Q., Li W., Feng J., Xie K. (2015). Carbon cycle in advanced coal chemical engineering. Chem. Soc. Rev..

[10] Song B., Zhang M., Teng Y., Zhang X., Deng Z., Huo L., Gao S. (2020). Highly selective ppb-level H_2_S sensor for spendable detection of exhaled biomarker and pork freshness at low temperature: Mesoporous SnO_2_ hierarchical architectures derived from waste scallion root. Sens. Actuators B Chem..

[11] Liu W., Tian K., He Y., Jiang H., Yu H. (2014). High-Yield Harvest of Nanofibers/Mesoporous Carbon Composite by Pyrolysis of Waste Biomass and Its Application for High Durability Electrochemical Energy Storage. Environ. Sci. Technol..

[12] Zhang L., Hu F., Zhu S., Lin Y., Meng Z., Yu R., Liu X. (2020). Meso-Reconstruction of Wool Keratin 3D “Molecular Springs” for Tunable Ultra-Sensitive and Highly Recovery Strain Sensors. Small.

[13] Zhang P., Zhang N., Wang Q., Wang P., Yuan J., Shen J., Fan X. (2019). Disulfide bond reconstruction: A novel approach for grafting of thiolated chitosan onto wool. Carbohydr. Polym..

[14] Macdermid-Watts K., Pradhan R., Dutta A. (2021). Catalytic Hydrothermal Carbonization Treatment of Biomass for Enhanced Activated Carbon: A Review. Waste Biomass Valoriz..

[15] Hu X., Nango K., Bao L., Li T., Hasan M.D.M., Li C. (2019). High Yields of Solid Carbonaceous Materials from Biomass. Green Chem..

[16] Snyder B.F. (2019). Costs of biomass pyrolysis as a negative emission technology: A case study. Int. J. Energy Res..

[17] Momodu D., Okafor C., Manyala N., Bello A., Zebazekana M.G., Ntsoenzok E. (2019). Transformation of Plant Biomass Waste into Resourceful Activated Carbon Nanostructures for Mixed-Assembly Type Electrochemical Capacitors. Waste Biomass Valoriz..

[18] Bi H., Yin Z., Cao X., Xie X., Tan C., Huang X., Chen B., Chen F., Yang Q., Bu X. (2013). Carbon Fiber Aerogel Made from Raw Cotton: A Novel, Efficient and Recyclable Sorbent for Oils and Organic Solvents. Adv. Mater..

[19] Zhao H., Cheng Y., Liu W., Yang L., Zhang B., Wang L., Ji G., Xu Z. (2019). Biomass-Derived Porous Carbon-Based Nanostructures for Microwave Absorption. Nano-Micro Lett..

[20] Bi Z., Kong Q., Cao Y., Sun G., Su F., Wei X., Li X., Ahmad A., Xie L., Chen C. (2019). Biomass-derived porous carbon materials with different dimensions for supercapacitor electrodes: A review. J. Mater. Chem. A.

[21] Liu H., Wu S., Tian N., Yan F., You C., Yang Y. (2020). Carbon foams: 3D porous carbon materials holding immense potential. J. Mater. Chem. A.

[22] Liu W., Jiang H., Yu H. (2015). Thermochemical conversion of lignin to functional materials: A review and future directions. Green Chem..

[23] Thompson E., Danks A.E., Bourgeois L., Schnepp Z. (2015). Iron-catalyzed graphitization of biomass. Green Chem..

[24] Shao L., Wu Z., Duan H., Talgar S. (2018). Discriminative and rapid detection of ozone realized by sensor array of Zn^2+^ doping tailored MoS_2_ ultrathin nanosheets. Sens. Actuators B Chem..

[25] He M., Lei J., Zhou C., Shi H., Sun X., Gao B. (2019). Growth of vertical MoS_2_ nanosheets on carbon materials by chemical vapor deposition: Influence of substrates. Mater. Res. Express.

[26] Livneh T., Sterer E. (2010). Resonant Raman scattering at exciton states tuned by pressure and temperature in 2H-MoS_2_. Phys. Rev. B.

[27] Li Y., Liu H., Wang X., Zhang X. (2019). Fabrication and performance of wool keratin/functionalized graphene oxide composite fibers. Mater. Today Sustain..

[28] Xie J., Zhang J., Li S., Grote F., Zhang X., Zhang H., Wang R., Lei Y., Pan B., Xie Y. (2013). Controllable Disorder Engineering in Oxygen-Incorporated MoS_2_ Ultrathin Nanosheets for Efficient Hydrogen Evolution. J. Am. Chem. Soc..

[29] Ahmad F., Mushtaq B., Butt F.A., Rasheed A., Ahmad S. (2021). Preparation and characterization of wool fiber reinforced nonwoven alginate hydrogel for wound dressing. Cellulose.

[30] Pandey S., Fosso-Kankeu E., Spiro M.J., Waanders F., Kumar N., Ray S.S., Kim J., Kang M. (2020). Equilibrium, kinetic, and thermodynamic studies of lead ion adsorption from mine wastewater onto MoS_2_-clinoptilolite composite. Mater. Today Chem..

[31] Alavijeh M.S., Maghsoudpour A., Khayat M., Rad I., Hatamie S. (2021). Cobalt ferrite decoration of molybdenum disulfide nanosheets; development of a nanocomposite-mediated hyperthermia method. J. Mech. Sci. Technol..

[32] Ferreira E.H., Moutinho M.V.O., Stavale F., Lucchese M.M., Capaz R.B., Achete C.A., Jorio A. (2010). Evolution of the Raman spectra from single-, few-, and many-layer graphene with increasing disorder. Phys. Rev..

[33] Zhang X., Han W., Wu J., Milana S., Lu Y., Li Q., Ferrari A.C., Tan P. (2013). Raman spectroscopy of shear and Layer Breathing Modes in Multilayer MoS_2_. Phys. Rev. B.

[34] Su S., Hsu Y.-T., Chang Y., Chiu C., Hsu C.-L., Hsu W.-T., Chang W., He H., Li L. (2014). Band gap-tunable molybdenum sulfide selenide monolayer alloy. Small.

[35] Cao P., Peng J., Liu S., Cui Y., Hu Y., Chen B., Li J., Zhai M. (2017). Tuning the Composition and Structure of Amorphous Molybdenum Sulfide/Carbon Black Nanocomposites by Radiation Technique for Highly Efficient Hydrogen Evolution. Sci. Rep..

[36] Sun Q., Wu Z., Duan H., Jia D. (2019). Detection of Triacetone Triperoxide (TATP) Precursors with an Array of Sensors Based on MoS_2_/RGO Composites. Sensors.

[37] Sun Q., Wu Z., Zhang M., Qin Z., Cao S., Zhong F., Li S., Duan H., Zhang J. (2021). Improved Gas-Sensitive Properties by a Heterojunction of Hollow Porous Carbon Microtubes Derived from Sycamore Fibers. ACS Sustain. Chem. Eng..

[38] Li J., Yang M., Cheng X., Zhang X., Guo C., Xu Y., Gao S., Major Z., Zhao H., Huo L. (2021). Fast detection of NO_2_ by porous SnO_2_ nanotoast sensor at low temperature. J. Hazard. Mater..

[39] Wu X., Qian C., Wu H., Xu L., Bu L., Piao Y., Diao G., Chen M. (2020). Gestated Uniform Yolk-Shell Sn@N-Doped Hollow Mesoporous Carbon Spheres with Buffer Space for Boosting Lithium Storage Performance. Chem. Commun..

[40] Wu H., Xia L., Ren J., Zheng Q., Xu C., Lin D. (2017). A high-efficiency N/P co-doped graphene/CNT@porous carbon hybrid matrix as cathode host for high performance lithium-sulfur batteries. J. Mater. Chem. A.

[41] Rao C.N.R., Gopalakrishnan K., Govindaraj A. (2014). Synthesis, properties and applications of graphene doped with boron, nitrogen and other elements. Nanotoday.

[42] Haridas V., Sukhananazerin A., Sneha J.M., Pullithadathil B., Narayanan B. (2020). α-Fe_2_O_3_ loaded less-defective graphene sheets as chemiresistive gas sensor for selective sensing of NH_3_. Appl. Surf. Sci..

[43] Su P.-G., Yang L. (2016). NH_3_ gas sensor based on Pd/SnO_2_/RGO ternary composite operated at room-temperature. Sens. Actuators B Chem..

[44] Wang Y., Zhang L., Hu N., Wang Y., Zhang Y., Zhou Z. (2014). Ammonia gas sensors based on chemically reduced graphene oxide sheets self-assembled on Au electrodes. Nanoscale Res. Lett..

[45] Klinbumrung A., Thongtem T., Phuruangrat A., Thongtem S. (2016). Optical and ammonia-sensing properties of SnO_2_ nanoparticles synthesized using a 900 W microwave. Jpn. J. Appl. Phys..

[46] Wang S., Ma J., Li Z., Su H., Alkurd N.R., Zhou W., Wang L., Du B., Tang Y., Ao D. (2015). Surface acoustic wave ammonia sensor based on ZnO/SiO_2_ composite film. J. Hazard. Mater..

[47] Fu T. (2013). CuS-doped CuO nanoparticles sensor for detection of H_2_S and NH_3_ at room temperature. Electrochim. Acta.

[48] Tohidi S., Parhizkar M., Bidadi H., Mohammad-Rezaei R. (2020). High-performance chemiresistor-type NH_3_ gas sensor based on three-dimensional reduced graphene oxide/polyaniline hybrid. Nanotechnology.

[49] Wang X., Gu D., Li X., Lin S., Zhao S., Rumyantseva M.N., Gaskov A.M. (2019). Reduced graphene oxide hybridized with WS_2_ nanoflakes based heterojunctions for selective ammonia sensors at room temperature. Sens. Actuators B Chem..

[50] Garg N., Kumar M., Kumari N., Deep A., Sharma A. (2020). Chemoresistive Room-Temperature Sensing of Ammonia Using Zeolite Imidazole Framework and Reduced Graphene Oxide (ZIF-67/rGO) Composite. ACS Omega.

[51] Wu Z., Zhou C., Zu B., Li Y., Dou X. (2016). Contactless and Rapid Discrimination of Improvised Explosives Realized by Mn^2+^ Doping Tailored ZnS Nanocrystals. Adv. Funct. Mater..

[52] Liu X., He J., Tang D., Liu Q., Wen J., Yu W., Lu Y., Zhu D., Liu W., Cao P. (2015). Band alignment of atomic layer deposited high-k Al_2_O_3_/multilayer MoS_2_ interface determined by X-ray photoelectron spectroscopy. J. Alloys Compd..

[53] Tao J., Yu X., Liu Q., Liu G., Tang H. (2021). Internal electric field induced S–scheme heterojunction MoS_2_/CoAl LDH for enhanced photocatalytic hydrogen evolution. J. Colloid Interface Sci..

[54] Zhang Z., Xu M., Liu L., Ruan X., Yan J., Zhao W., Yun J., Wang Y., Qin S., Zhang T. (2018). Novel SnO_2_@ZnO hierarchical nanostructures for highly sensitive and selective NO_2_ gas sensing. Sens. Actuators B Chem..

