# Supplementary material for: Wool-Based Carbon Fiber/MoS2 Composite Prepared by Low-Temperature Catalytic Hydrothermal Method and Its Application in the Field of Gas Sensors
Source: Nanomaterials (Basel). 2022 Mar 28;12(7):1105. doi: 10.3390/nano12071105 (PMC9000424; doi:10.3390/nano12071105)
Supplement: Supplementary file 1 [file nanomaterials-12-01105-s001.zip › nanomaterials-1608644-supplementary.pdf]

Wool-Based Carbon Fiber/MoS_2 Composite Prepared by Low-Temperature Catalytic Hydrothermal Method and Its Application in the Field of Gas Sensors

Yidan Xia [1], Zhaofeng Wu [1,*], Zhangjie Qin [2], Fengjuan Chen [2], Changwu Lv [2], Min Zhang [2], Talgar Shaymurat [3] and Haiming Duan [1,2]

1 Xinjiang Key Laboratory of Solid State Physics and Devices, Xinjiang University, Urumqi 830046, China; xiayidaaa@163.com (Y.X.); dhm@xju.edu.cn (H.D.)
2 School of Physics Science and Technology, Xinjiang University, Urumqi 830046, China; qinzj0725@163.com (Z.Q.); fjchen@xju.edu.cn (F.C.); lvchw@xju.edu.cn (C.L.); minzhang0816@163.com (M.Z.)
3 Key Laboratory of New Energy and Materials Research, Xinjiang Institute of Engineering, Urumqi 830023, China; talgar.shaymurat@vip.163.com
* Correspondence: wuzf@xju.edu.cn

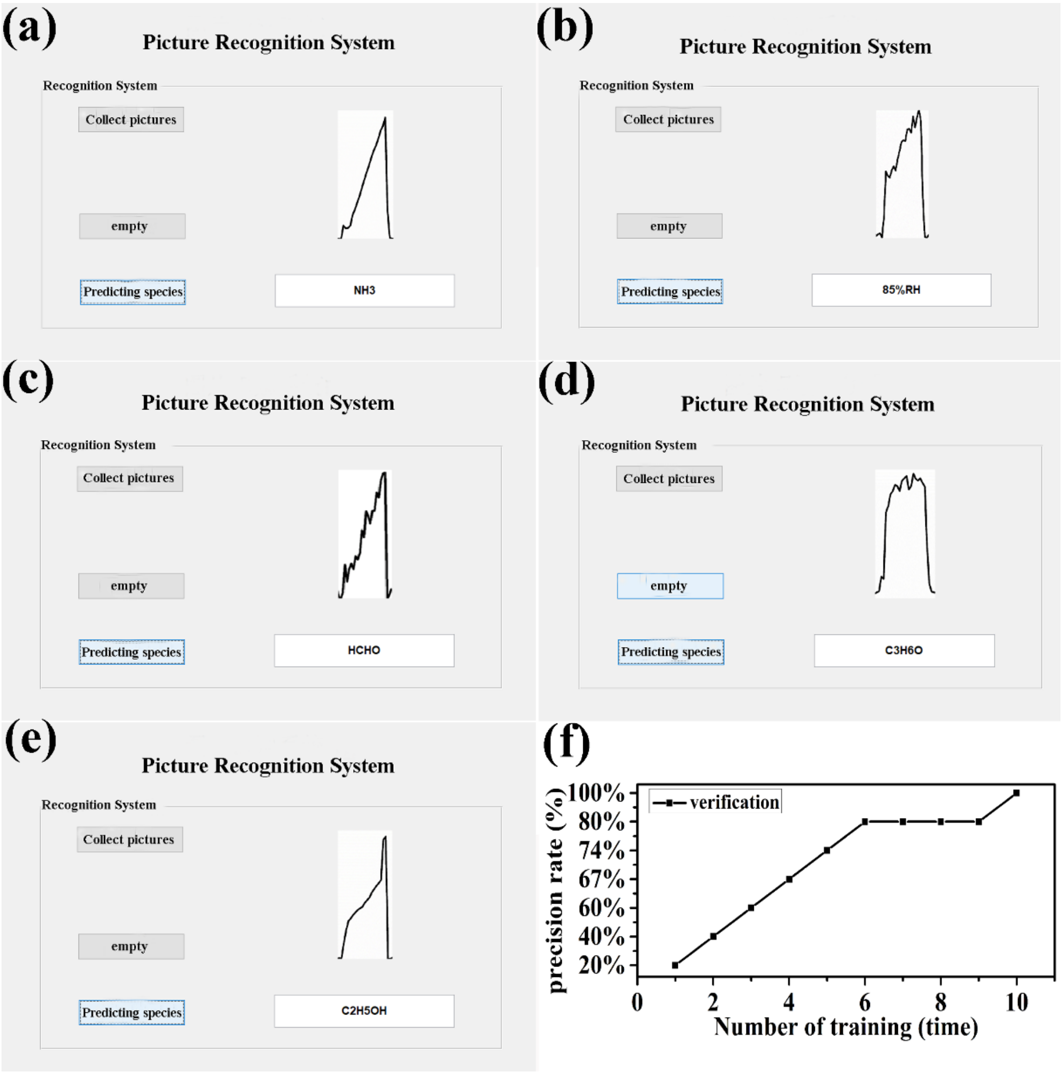

Figure S1. Identification of (**a**) NH_3, (**b**) 85% RH, (**c**) HCHO, (**d**)C_3H_6O and (**e**)C_2H_5OH target analytes by MATLAB software, (**f**) Curve of training times and accuracy

Inspired by the WCF-MoS_2 sensor producing different sensing curves for five analytes, we use image recognition technology to help improve the recognition and detection of the target atmosphere. The model was established by MATLAB software, and the five gases were identified. The three peaks corresponding to each gas will be made into a map respectively, and finally two of the three maps for each gas will be selected as the database. The machine builds the model by learning these two pictures, and finally uses the remaining picture for verification. After the Picture Recognition System is established, click on the collected picture to select the picture to be recognized and import it in, and finally click on the prediction type option, and the detection result will appear in the lower right (**Figure S1 a-e**). As shown by **Figure S1f**, with the increase of learning times, the recognition accuracy based on software is also increasing synchronously. When the machine learning reaches 10 times, the recognition accuracy of five analytes reaches 100%.
